# EphA2-Induced Angiogenesis in Ewing Sarcoma Cells Works through bFGF Production and Is Dependent on Caveolin-1

**DOI:** 10.1371/journal.pone.0071449

**Published:** 2013-08-12

**Authors:** Miguel Sáinz-Jaspeado, Juan Huertas-Martinez, Laura Lagares-Tena, Juan Martin Liberal, Silvia Mateo-Lozano, Enrique de Alava, Carmen de Torres, Jaume Mora, Xavier Garcia del Muro, Oscar M. Tirado

**Affiliations:** 1 Sarcoma Research Group, Laboratori d’Oncología Molecular, Institut d’Investigació Biomèdica de Bellvitge (IDIBELL), L’Hospitalet de Llobregat, Barcelona, Spain; 2 Nanomedicine Research Program, Molecular Biology and Biochemistry Research Center, CIBBIM-Nanomedicine, Vall d'Hebron Hospital Research Institute, Barcelona, Spain; 3 Centro de Investigación del Cáncer-IBMCC (University of Salamanca-CSIC), and University Hospital of Salamanca, Salamanca, Spain; 4 Developmental Tumor Biology Laboratory, Hospital Sant Joan de Deu, Barcelona, Spain; Sun Yat-sen University Medical School, China

## Abstract

Angiogenesis is the result of the combined activity of the tumor microenvironment and signaling molecules. The angiogenic switch is represented as an imbalance between pro- and anti-angiogenic factors and is a rate-limiting step in the development of tumors. Eph receptor tyrosine kinases and their membrane-anchored ligands, known as ephrins, constitute the largest receptor tyrosine kinase (RTK) subfamily and are considered a major family of pro-angiogenic RTKs. Ewing sarcoma (EWS) is a highly aggressive bone and soft tissue tumor affecting children and young adults. As other solid tumors, EWS are reliant on a functional vascular network for the delivery of nutrients and oxygen and for the removal of waste. Based on the biological roles of EphA2 in promoting angiogenesis, we explored the functional role of this receptor and its relationship with caveolin-1 (CAV1) in EWS angiogenesis. We demonstrated that lack of CAV1 results in a significant reduction in micro vascular density (MVD) on 3 different *in vivo* models. *In vitro,* this phenomenon correlated with inactivation of EphA2 receptor, lack of AKT response and downregulation of bFGF. We also demonstrated that secreted bFGF from EWS cells acted as chemoattractant for endothelial cells. Furthermore, interaction between EphA2 and CAV1 was necessary for the right localization and signaling of the receptor to produce bFGF through AKT and promote migration of endothelial cells. Finally, introduction of a dominant-negative form of EphA2 into EWS cells mostly reproduced the effects occurred by CAV1 silencing, strongly suggesting that the axis EphA2-CAV1 participates in the promotion of endothelial cell migration toward the tumors favoring EWS angiogenesis.

## Introduction

Caveolin-1 (CAV1) is an integral membrane protein involved in caveolae biogenesis, cholesterol homeostasis, intracellular trafficking and signal transduction, amongst other functions. The precise role of caveolin-1 in tumorigenesis and whether the protein acts as a tumor suppressor or as an oncogene seems to be cell type and context-dependent [1]. Recently, we identified *CAV1* as a metastasis-associated gene that is a transcriptional target of EWS/FLI1 as well as an important determinant of Ewing sarcoma (EWS) malignant phenotype and tumorigenicity [2]. CAV1 has been shown to generate autocrine and paracrine positive feedback loops by increasing several factors mRNA stability, leading to increased levels of these proteins and increased pro-angiogenic activities [3]. However, this potential role has not been explored yet in EWS.

The process of tumor neovascularization proceeds through the combined output of inductive signals from the entire cellular constituency of the tumor. The concept of the “angiogenic switch,” whereby tumors acquire the ability to grow exponentially and disseminate beyond their primary site, is one of the central components in our understanding of cancer [4]. The new vessels not only help to meet the growing metabolic demands of the tumor by supplying additional nutrients, but also provide potential routes for tumor dissemination and metastasis [5]. During the initial phase, genetic changes and local hypoxia in tumors contribute to increased secretion of soluble angiogenic factors by tumor cells, stromal cells and inflammatory cells. These angiogenic factors include: vascular endothelial growth factor (VEGF), basic fibroblast growth factor (bFGF), platelet derived growth factor (PDGF), epidermal growth factor (EGF), insulin-like growth factor (IGF), placental growth factor (PlGF), and others. All these factors promote the sprouting of new vessels from nearby existing vessels [6].

Eph receptor tyrosine kinases and their membrane-anchored ligands, known as ephrins, constitute the largest receptor tyrosine kinase (RTK) subfamily, including at least 16 receptors and 9 ligands in vertebrates [7]. Based on their binding preference to one or the other class of ephrins, Eph receptors have been subdivided into two subclasses, EphA and EphB. Eph receptors have diverse activities, including widespread effects on the intercellular junctions, cell shape, cell-substrate adhesion, cell movement and angiogenesis [8]. Moreover, the ephrin family is considered to be a major family of pro-angiogenic RTKs. Of these molecules, the EphA2 receptor, initially linked with neuronal migration during embryogenesis [9], is the most thoroughly studied with regard to its role in angiogenesis, and it has been implicated in responses such as endothelial cell migration and vascular assembly as well as regulation of epithelial cell junctions [10].

EWS is a highly aggressive bone and soft tissue tumor affecting children and young adults [11]. Patients most commonly die as a result of the development of metastases [12]. Abnormal vessel growth and function are hallmarks of cancer disease, and they contribute to metastasis and disease progression [13]. EWS, like other solid tumors, are reliant on a functional vascular network for the delivery of nutrients and oxygen and for the removal of waste [14]. Upon clinical presentation, Ewing tumors are highly hemorrhagic and harbor large and friable vessels. Therefore, defining the molecular mechanisms that direct blood vessel formation in EWS is key identifying new therapeutic targets that might prevent vascular development and metastasis.

Based on the biological roles of EphA2 in promoting angiogenesis [15], we explored the functional role of this receptor and its relationship with CAV1 in EWS angiogenesis. We demonstrated that lack of CAV1 results in a significant reduction in micro vascular density (MVD) in 3 different *in vivo* models. *In vitro,* this phenomenon correlated with inactivation of EphA2 receptor and downregulation of bFGF. We also demonstrated that secreted bFGF from EWS cells acted as chemoattractant for endothelial cells. Furthermore, interaction between EphA2 and CAV1 was necessary for the right localization and signaling of the receptor to produce bFGF through AKT. Finally, introduction of a dominant-negative form of EphA2 into EWS cells reproduced most of the effects occurred by CAV1 silencing, strongly suggesting that the axis EphA2-CAV1 participates in the promotion of endothelial cell migration toward the tumors favoring EWS angiogenesis.

## Results

### Growth Reduction in CAV1 Knocked down Xenografts is a Consequence of Impaired Angiogenesis

**Figure 1:**
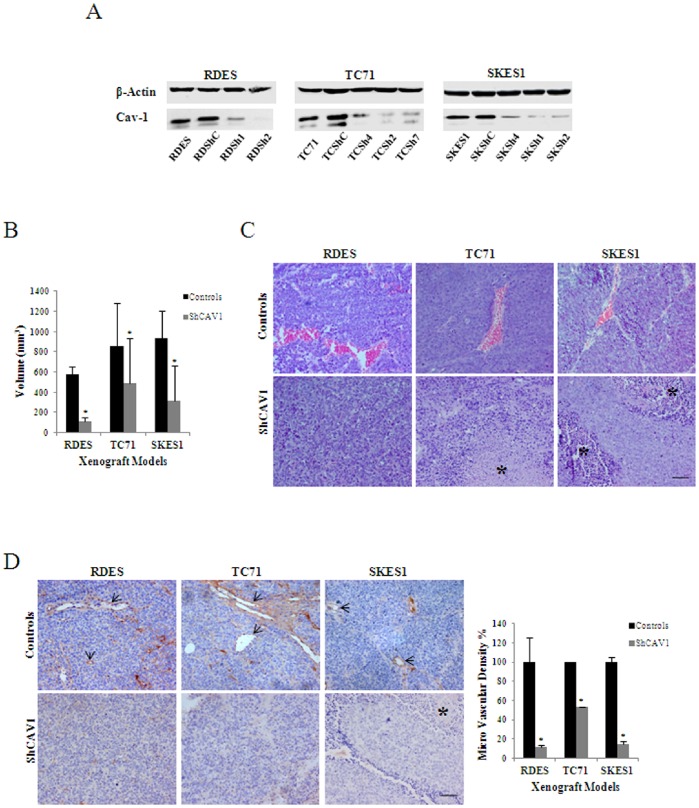
CAV1 silencing reduces vascularization in EWS. **A.** Immunoblot showing substantially reduced CAV1 levels in selected clones of RDES, TC71 and SKES1 cells stably expressing a CAV1 shRNA compared with untransfected cells and cells transfected with an empty vector. β-Actin was used as loading control. **B.** Mice xenograft volumes comparing tumors from grouped control cells (parental n = 5; empty vector n = 5) and 2 CAV1 knocked-down cells (n = 5 for every clone); bars, SD (*P≤0.05) **C.** Hematoxylin and Eosin (H&E) staining from corresponding paraffin-embedded tumors. Asterisks indicate necrotic lakes Scale bar, 50 µm. **D.** CD31 immunohistochemistry detecting positive blood vessels in paraffin-embedded tumors. Arrows indicate blood vessel staining Scale bar, 50 µm. Graph shows MVD comparing all three models and showing the constant reduction of blood vessels in CAV1 knocked-down cells; bars, SD (*P≤0.05).

To further explore the mechanism by which CAV1 promotes tumor growth in EWS, we knocked down CAV1 expression in RDES, TC71 and SKES1 cells (Figure 1A) as previously described [2], [16]. In agreement with previous results [2], CAV1 silencing resulted in significant tumor growth reduction in all three models (P≤0.05) (Figure 1B). H&E staining showed a reduced number of vessels and a major incidence of necrosis in tumor sections from CAV1 knocked down cells (Figure 1C). Moreover, CD31 staining showed a significant reduction on MVD (P≤0.05) in CAV1 knocked down tumors (Figure 1D).

**Figure 2:**
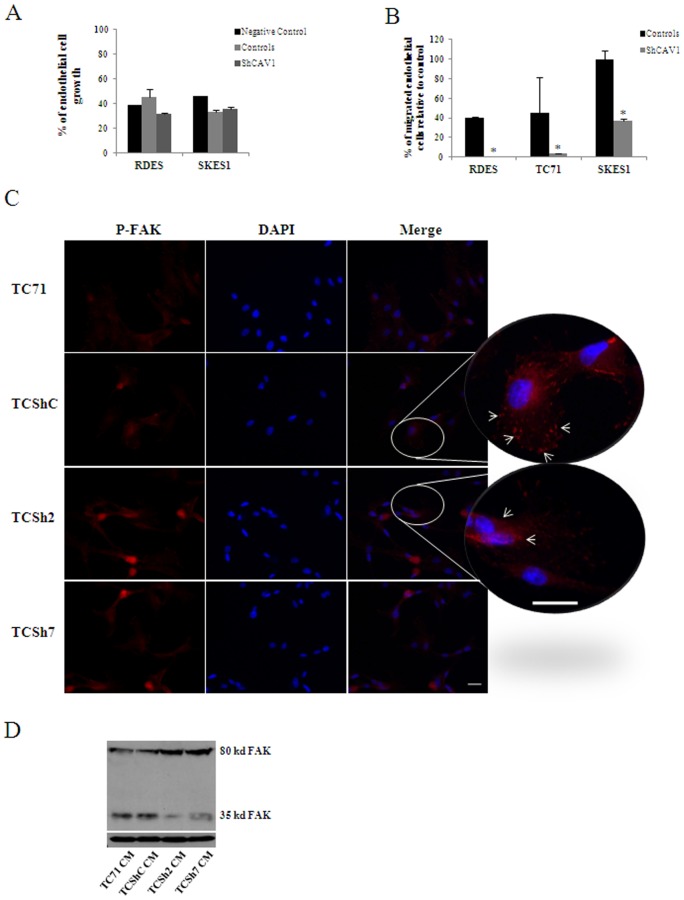
CAV1 silencing results on reduced endothelial cell migration. **A.** Quantification of endothelial cell growth comparing conditioned media (CM) from CAV1 knocked-down models versus RPMI without serum as negative control (n = 3). **B.** Quantification of endothelial cell migration where CM from RDES and TC71 models was used as chemoattractant for endothelial cells; bars, SD (*P≤0.05 n = 3) **C.** Phospho-FAK immunofluorescence. Endothelial cells cultured with CM from TC71 CAV1 knocked-down model, showing a decrease on phospho-FAK sprouts. Scale bar, 20 µm. **D.** FAK expression was analyzed in PAEC cells grown in CM from the TC71 model. Blot shows higher expression of 35 kd cleaved FAK form in cells grown with TC71 control CM than with TC71 Sh CM.

**Figure 3:**
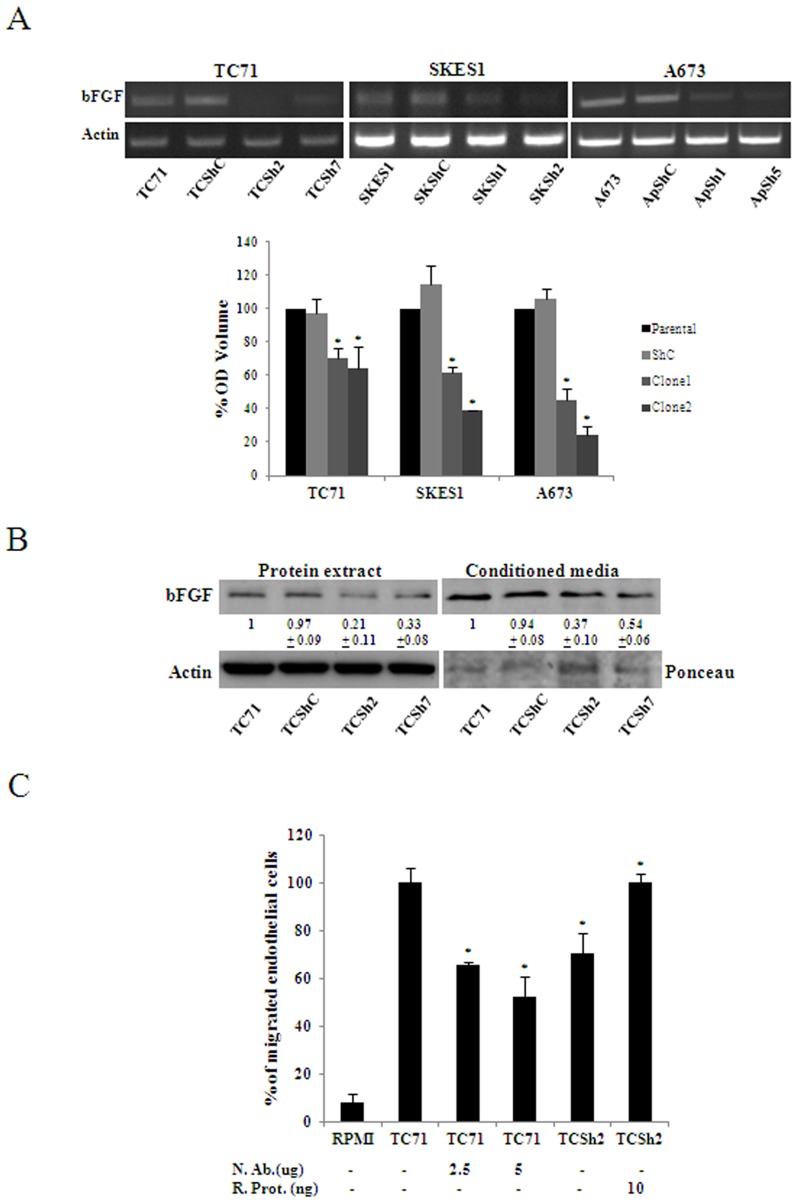
CAV1 silencing results in reduced bFGF transcription and secretion. **A.** bFGF expression was analyzed in CAV1 knocked-down models showing that bFGF expression was affected constantly in all models. The graph shows the quantification of the OD volume determined from the intensity of the bands from the RT-PCR images showing the statistically significant reduction in bFGF in CAV1 knocked-down cells; bars, SD (*P≤0.05 n = 3) **B.** Immunoblot showing bFGF reduction in the TC71 model either in total protein extract or CM. Actin blot and ponceau staining was used as loading control. Quantitative data measuring relative intensity of the bands is indicated below each lane (n = 3) **C.** Quantification of endothelial cell migration. CM was used as chemoattractant for endothelial cells. A neutralizing antibody -N. Ab- (2.5 µg/ml and 5 µg/ml) and a recombinant protein -R. Prot- (10 ng/ml); bars, SD (*P<0.002– when compared with parental CM – and *P = 0.0005– when compared with clone n = 3) were used to block and promote migration respectively. RPMI media alone was used as negative control.

Tumor cells secrete factors that promote the growth and the migration of endothelial cells to the tumor [17]. Conditioned media (CM) from CAV1 knocked down cells showed significant reduced capability to promote migration of endothelial cells (P≤0.05) with no changes in proliferation (Figure 2A–B). Moreover, immunofluorescence of phospho-FAK in endothelial cells grown with CM from control cells showed its localization on sprouts. In contrast, in endothelial cells grown with CM from CAV1 knocked down cells phosphorylated FAK appeared in a more perinuclear location (Figure 2C). Analysis of FAK expression showed higher levels of cleaved FAK in those cells grown with CM from control cells (Figure 2D). These results suggested that CM from CAV1 knocked down cells had reduced levels or lack of a factor that promoted migration of endothelial cells. In order to ascertain which factor or factors may be responsible for such activity, we explored by RT-PCR the expression of several pro-angiogenic factors in different EWS cell lines (Figure S1). Of all pro-angiogenic factors tested, bFGF was the only one reduced upon CAV1 knock down in all three models (Figure 3A and Figure S2). Accordingly, in the TC71 model, protein expression was also reduced from cell extracts and in CM (Figure 3B). Furthermore, CM from parental TC71 cells in the presence of a neutralizing antibody against bFGF showed similar reduced capability promoting endothelial cell migration than CAV1 knocked down cells (Figure 3C). Addition of recombinant bFGF to CM from CAV1 knocked down cells restored endothelial cell migration (Figure 3C) strongly suggesting that bFGF secreted from EWS cells promotes endothelial cell migration.

### Proper Localization and Signaling of EphA2 Receptor Depends on CAV1 Interaction

**Figure 4:**
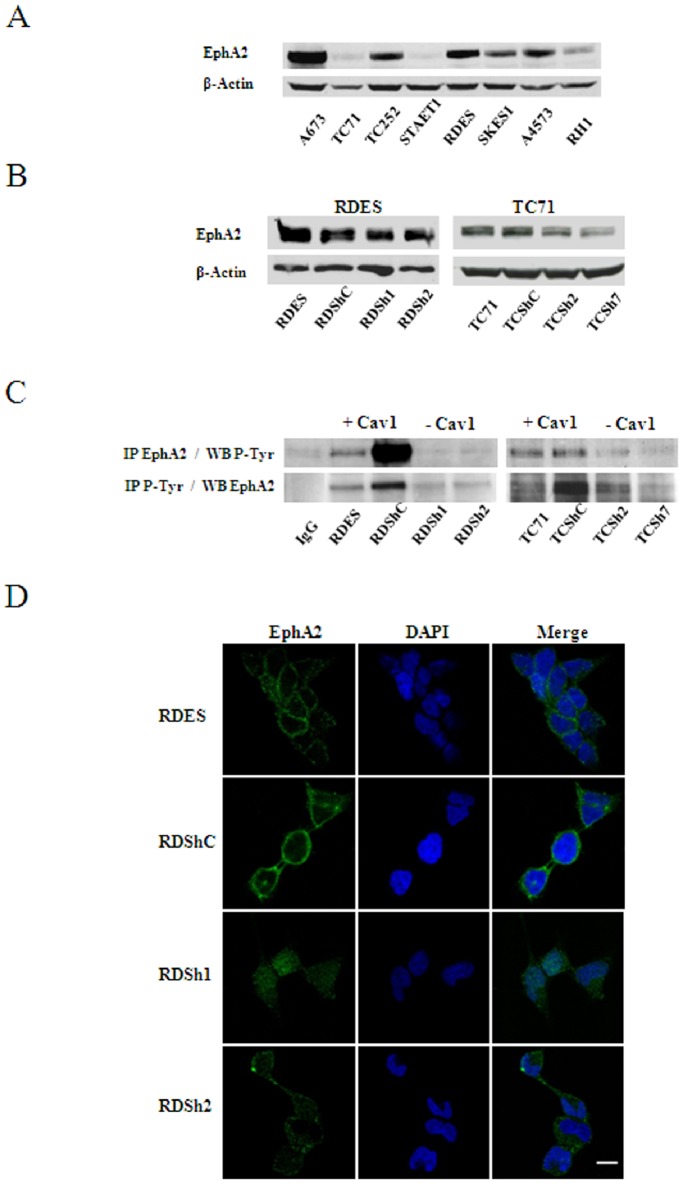
EphA2 receptor is expressed in EWS cells and is constitutively activated. **A.** Immunoblot of EphA2 in EWS cell lines **B.** Immunoblot of EphA2 in CAV1 knocked-down models showing a slight reduction of EphA2 expression in knocked-down cells when compared with parental and control cells. **C.** Immunoprecipitation of EphA2 blotted with phosphotyrosine and immunoprecipitation of phosphotyrosine blotted with EphA2 confirming its constitutive activation. **D.** Immunofluorescence of the RDES model showing EphA2 redistribution in cell cytoplasm as a consequence of CAV1 silencing. DAPI was used to identify the nucleus. Scale bar, 10 µm.

**Figure 5:**
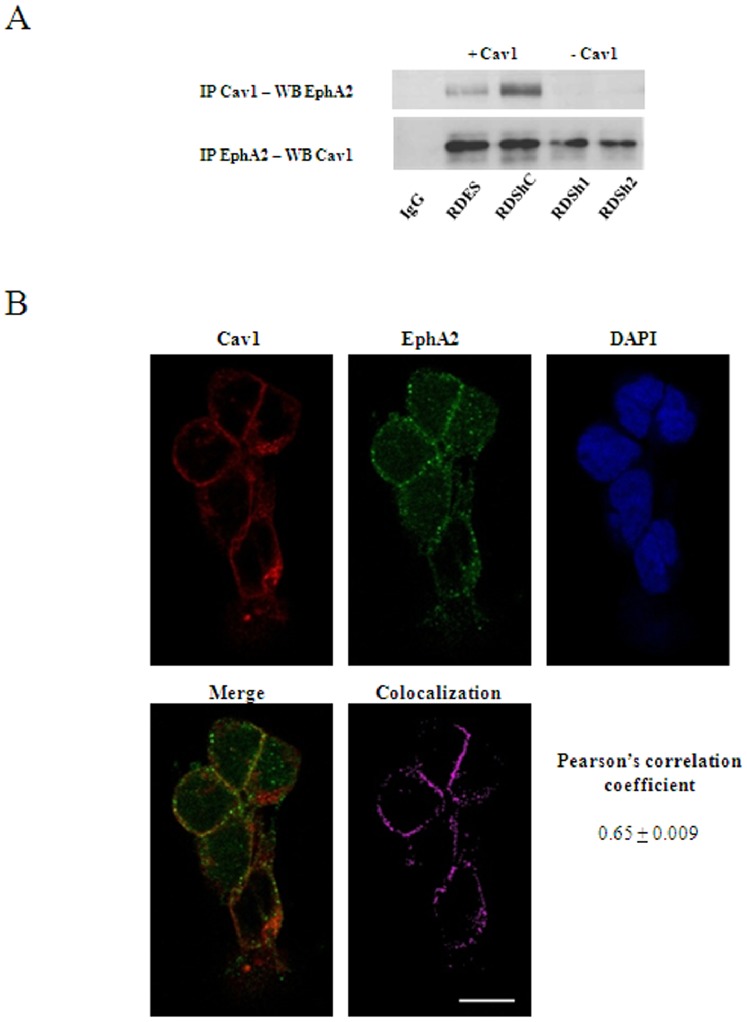
CAV1 interacts with EphA2 receptor. **A.** Co-immunoprecipitation of CAV1 and EphA2 showing their interaction. **B.** Co-immunofluorescence of CAV1 and EphA2 showing co-localization in cell membrane in RDES cells. DAPI was used to identify the nucleus. The grade of co-localization was measured by the Pearson’s correlation coefficient (PCC) PCC = 0.65±0.009. Scale bar, 10 µm.

**Figure 6:**
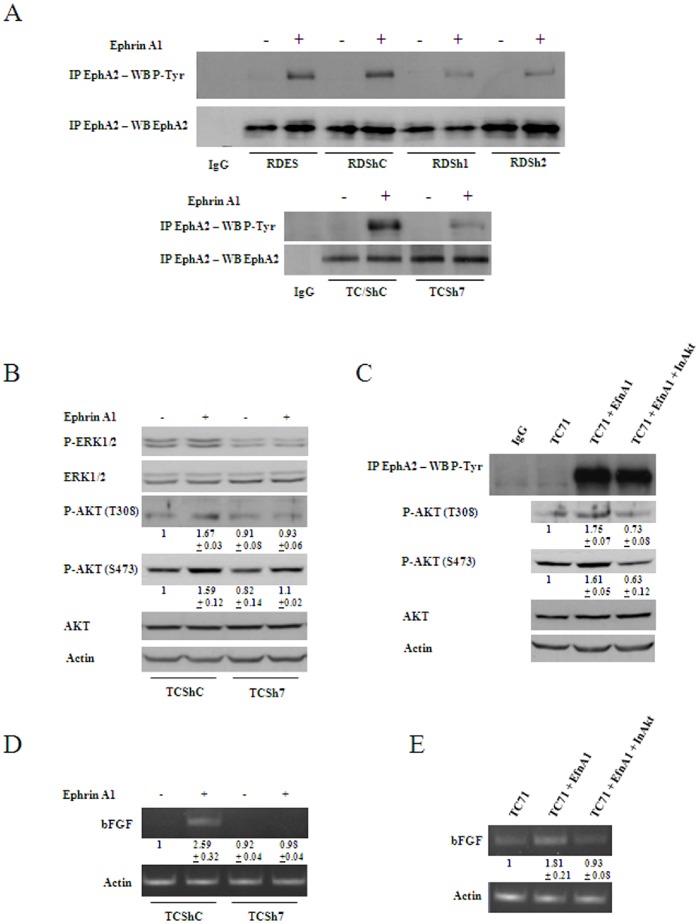
Stimulation of EphA2 promotes bFGF transcription through activation of AKT. **A.** Immunoblot showing higher EphA2 phosphorylation in response to ephrin-A1 stimulation in control cells compared with CAV1 knocked down starved cells. EphA2 blotting was used as control. **B.** Immunoblot showing activation of AKT in control cells and RT-PCR showing higher bFGF transcription after ephrin-A1 stimulation. No significant changes on ERK1/2 phosphorylation are shown. Quantitative data measuring relative intensity of the bands is indicated below each lane for the phospho-AKTs western analysis (n = 5) **C.** Immunoblot showing blockage of AKT activation in TC71 cells stimulated with ephrin-A1 in the presence of an AKT inhibitor (5 µM). Quantitative data measuring relative intensity of the bands is indicated below each lane (n = 5). **D and E.** By RT-PCR bFGF transcript changes are shown. Quantitative data measuring relative intensity of the bands is indicated below each lane (n = 3).

CAV1 has been shown to function as an activator of EphA2 signaling in endothelial cells [18]. Thus, we sought whether EphA2 was expressed in EWS. As shown in figure 4A, EphA2 receptor was widely expressed among EWS cell lines. Also, immunohistochemical analysis of a panel of 28 patients showed high expression of EphA2 in all samples. Moreover, although EWS cells lines showed expression of other members of the Eph receptor family (data not shown), their expression in tumor samples was not observed (Figure S3). Interestingly, CAV1 silencing resulted in a slight, if any, decrease of EphA2 protein expression (Figure 4B), a clear decrease on its phosphorylation (Figure 4C) and a displacement of the receptor from the membrane to the cytosolic fraction of the cell (Figure 4D). Of note, in EWS cells EphA2 receptor was shown to be constitutively phosphorylated (Figure 4C) suggesting that EWS cells express its main ligand ephrin-A1. As shown in Figure S4, EWS cells indeed express this ligand. Based on the results exposed above we explored the possibility that CAV1 was promoting localization and signaling of EphA2 through direct interaction in EWS cells. Consequently, cell lysate from the RDES model was subjected to immunoprecipitation using specific antibodies. As seen in Figure 5A, EphA2 was present in the CAV1 immunoprecipitated samples and vice versa, suggesting that CAV1 interacts with EphA2. No fusion proteins were detected in control immunoprecipitation reactions performed with IgG antibodies (Figure 5A). Accordingly, confocal microscopy analysis showed co-localization of both proteins in the plasma membrane of RDES cells (Figure 5B) and xenografts (Figure S5). In order to further demonstrate the implication of CAV1 in EphA2 signaling we stimulated cells from the RDES and TC71 models with a soluble activating form of ephrin-A1 (ephrin-A1 Fc). Contrary to CAV1 knocked down cells, addition of ephrin-A1 to controls resulted in tyrosine hyper-phosphorylation of EphA2 (Figure 6A). Interestingly, after ephrin stimulation no activation of ERKs was observed in the whole model. However, it resulted in activation of AKT (Figure 6B) and overexpression of bFGF (Figure 6D) in control cells but not in CAV1 knocked down cells, suggesting that the interaction of CAV1 with EphA2 in the presence of the ligand activates AKT signaling to promote bFGF expression. To further demonstrate the implication of AKT in producing bFGF we stimulated TC71 cells with ephrin-A1 in the presence of an AKT inhibitor. Results showed that cells stimulated in the presence of the AKT inhibitor, although showing stimulation of EphA2 (Figure 6C), did not respond inducing more bFGF transcript (Figure 6E). These results strongly suggest that, indeed, induction of bFGF after ephrin-A1 stimulation occurs through activation of the AKT signaling.

### Promotion of Angiogenesis through EphA2 is Kinase-dependent

**Figure 7:**
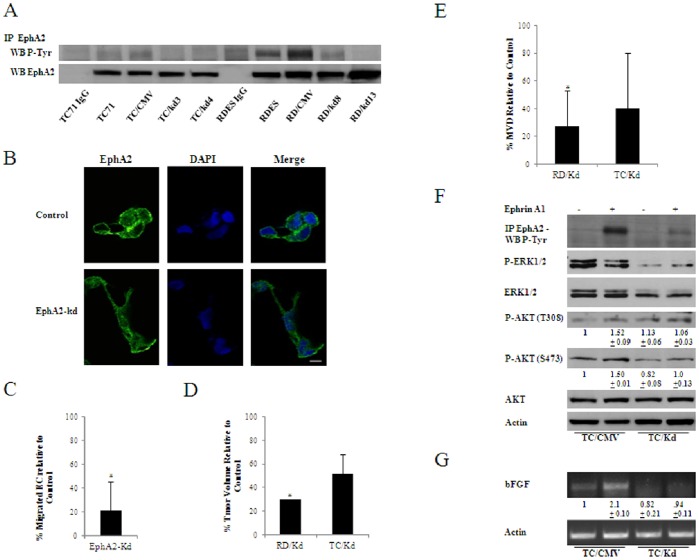
Dominant negative EphA2 recapitulates CAV1 silencing effects on angiogenesis. **A.** Phosphotyrosine immunoblot confirming the dominant negative effects of EphA2-Kd vector in RDES and TC71 cells (RD/CMV and TC/CMV states for empty-vector transfected cells). EphA2 blotting was used as control. **B.** Immunofluorescence of EphA2 showing redistribution in cell cytoplasm as a consequence of EphA2-Kd transfection. Scale bar, 10 µm. **C.** Quantification of transwell cell migration, CM from the EphA2-Kd models was used as chemoattractant for endothelial cells; bars, SD (*P≤0.05 n = 3); vertical bars represent mean percentage of migrated endothelial cells relative to control. **D.** Mice xenograft volumes comparing tumors of 2 EphA2-Kd cell clones (n = 5 for each clone) relative to grouped controls (parental n = 5; empty vector n = 5); vertical bars represent mean percentage of tumor volumes relative to controls; bars, SD (*P≤0.05) **E.** MVD comparing both EphA2-Kd models and showing a reduction of blood vessels in selected clones relative to controls; bars, SD (*P≤0.05) **F.** Immunoblot of the TC71 model (Control and EphA2-Kd clone) stimulated with ephrin-A1 showing no changes on the ERK1/2 pathway and activation of AKT. Quantitative data measuring relative intensity of the bands is indicated below each lane for the phospho-AKTs western analysis (n = 5) **G.** RT-PCR showing increased expression of bFGF in control cells compared to EphA2-Kd cells after EphA2 stimulation. Quantitative data measuring relative intensity of the bands is indicated below each lane (n = 3).

Forward EphA2 signaling is mainly kinase-dependent. Nonetheless, Eph receptors are nonclassical receptor tyrosine kinases because, besides kinase-dependent signaling, ligation of certain members of the Eph family can also trigger kinase-independent responses [19]. Given that CAV1 silencing affected tyrosine phosphorylation of EphA2, we asked whether disrupting EphA2 kinase-dependent activities by stably transfecting a dominant-negative construct of EphA2 (EphA2-kd) into RDES and TC71 cells would recapitulate the anti-angiogenic effects derived from CAV1 silencing. Similar to CAV1 knocked down cells EphA2-kd cells in both models showed a clear decrease in constitutive EphA2 phosphorylation (Figure 7A) and a displacement of the receptor from the membrane to the cytosolic fraction of RDES cells (Figure 7B). Also, conditioned media from RDES EphA2-kd cells showed significant reduced capability to promote migration of endothelial cells (P≤0.05) (Figure 7C). Tumor growth reduction was significant in the RDES EphA2-kd model (P≤0.05) and considerable (about 50%) although not reaching significance (P = 0.19) in the TC71 EphA2-kd model (Figure 7D). Histological analysis of tumor sections however, showed considerable higher amounts of necrosis in EphA2-kd cells from both models (Figure S6). Accordingly, MVD reduction was significant in the RDES model (P≤0.05) and considerably lower (40%) in the TC71 model (Figure 7E). Lastly, after ephrin stimulation EphA2-kd cells from the TC71 model neither showed AKT activation (Figure 7F) nor overexpression of bFGF (Figure 7G) suggesting that EphA2 kinase-dependent signaling through activation of AKT is necessary for bFGF overexpression and promotion of angiogenesis in EWS tumors.

## Discussion

Like all solid tumors, EWS require a viable vascular supply for tumor cells to grow beyond the limits of oxygen and nutrient diffusion into tissues [14]. EWS basically rely on a combination of angiogenesis and vasculogenesis [20]. Apart from what is known about the role of vasculogenesis in EWS vascular tumor blood supply [20], little is known about the regulation of angiogenesis sprouting. As shown previously, CAV1 silencing in EWS cells impairs tumor growth in nude mice [2]. Because CAV1 has been shown to contribute to the progress of angiogenesis in different tumor entities [21], [22], we sought to determine whether the impairment of tumor growth correlated with impaired angiogenesis as a consequence of CAV1 silencing and the mechanisms involved. We established three new models of low CAV1 expression in EWS cells. In all three, reduction of tumor growth correlated with a significant decrease of MVD. To our knowledge, this is the first study demonstrating a direct effect on tumor angiogenesis by knocking down CAV1 in tumor cells.

In order to determine the effects of EWS cell CAV1 silencing on endothelial cells, we used CM from the models and compared, *in vitro*, the proliferative and migratory capacity of endothelial cells. Results showed that CM from knocked down cells had a reduced capability to induce endothelial cell migration without affecting proliferation. Activation of FAK has been shown during endothelial cell migration [23], [24]. Moreover, FAK proteolysis has been demonstrated to correlate with adhesion dynamics and migration [25]. Accordingly, activation and higher proteolysis of FAK was observed only in endothelial cells in the presence of control conditioned media. These results suggested that CAV1 silencing negatively affected the expression or production of factors related to endothelial cell migration.

EWS cells express a variety of pro-angiogenic factors [14], [20], [26]. Analysis of the levels of a group of pro-angiogenic factors released by tumor cells showed that only the expression of the *bFGF* gene was reduced by CAV1 silencing. In agreement with our results, VEGF has been shown to be expressed in EWS cells [27] and to promote vasculogenesis. Furthermore, *VEGF* has been postulated to be an indirect target of EWS/FLI1 [28]. VEGF released from tumor cells is the most important pro-angiogenic factor that stimulates both sprouting and proliferation of endothelial cells. Nevertheless, other factors such as PDGF, FGF and PlGF are also important [29]. One of the most important roles of bFGF is promoting endothelial cell migration [30], [31]. This would explain why the major effect observed in endothelial cells as a consequence of CAV1 silencing in tumor cells was reduced migration. Moreover, experiments performed with exogenous bFGF and its neutralizing antibody further supported the importance of bFGF in endothelial cell migration. Importantly, increases in bFGF expression can enhance tumor angiogenesis and promote tumor growth [32]. However, as a complicated multistep process, tumor-associated angiogenesis is implicated in the extensive interplay among cells, soluble factors, and extracellular matrix (ECM) components. Moreover, the “angiogenic switch” via the change of the local balance of pro- and anti-angiogenic factors, is critical for the initiation of tumor angiogenesis [33]. Thus, we cannot rule out the possibility that CAV1 also plays an important role in the regulation of other complicated processes in angiogenesis.

The role of eph receptors and ephrins in tumor angiogenesis is well acknowledged [34]. Expression of several eph receptor and ephrin family members in EWS cells has been shown previously [35]. Ephrin A1, EphA2, EphB3 and EphB4 expression was observed in our panel of EWS cell lines. However, only EphA2 was seen in tumor samples. This is of relevance because ours is the first study demonstrating the expression of EphA2 protein in EWS patient samples. Eph receptors contain a caveolin-binding motif within their kinase domain (WSYGIVMW for EphA2). Moreover, interaction of EphA2 with CAV1 has been shown as key for proper signaling and membrane targeting of the receptor [18]. By a combination of immunoprecipitation and confocal microscopy assays we show that, indeed in EWS cells CAV1 interacts with EphA2. Furthermore and in agreement with previous results [18], CAV1 silencing resulted in delocalization of the receptor and moderate decrease in its expression suggesting some sort of degradation after losing its targeting to the membrane. Degradation of eph receptors, as for other receptors, has been shown after endocytosis following activation [36]. Nonetheless, this might be not the case because constitutive tyrosine phosphorylation of EphA2 in EWS cells is lost after CAV1 silencing. However, whether this mere loss of protein depends on degradation or not, remains to be further determined.

In order to establish a relationship between EphA2 activation and bFGF expression we decided to perform EphA2 stimulation assays with ephrin-A1. As expected [18], CAV1 knocked down cells responded less efficiently to ephrin-A1 stimulation than control cells. EphA2 signaling has been repeatedly shown to occur through MAPKs [37], [38]. AKT signaling has also been shown to be activated as a consequence of EphA2 stimulation in several cell lines [39]. In our model, stimulation of EphA2 by its ligand resulted in an increase of AKT phosphorylation and higher transcription of bFGF. However, no activation of MAPK signaling was observed. Lack of increased bFGF transcription in stimulated cells with the presence of an AKT inhibitor, that inhibits AKT phosphorylation without affecting PI3K, further confirmed the involvement of AKT signaling in bFGF transcription in response to EphA2 stimulation.

EphA2 kinase is frequently up-regulated in several different types of cancer [40]. It is not clear whether EphA2 phosphorylation or its overexpression plays a role in tumor progression. In fact, simply overexpression of the receptor likely will lead to Eph kinase-independent responses [41]. Because CAV1 silencing affected primarily EphA2 tyrosine phosphorylation we focused on kinase-dependent activities. In agreement with studies performed in prostate cancer cells [41], introduction of a dominant negative form of EphA2 into TC71 and RDES recapitulated most of the effects induced by CAV1 silencing. Thus, we observed a decrease of constitutive EphA2 phosphorylation and the consequent displacement of EphA2 receptor from the membrane to the cytosolic fraction in RDES cells. Conditioned media from RDES EphA2-kd cells showed reduced capability to promote migration of endothelial cells. Furthermore, tumor growth reduction was observed in both models showing a great degree of necrosis. Accordingly, MVD reduction was also observed in both models. Lastly, after ephrin stimulation EphA2-kd cells from the TC71 model neither showed AKT activation nor overexpression of bFGF. Overall, our results underscore the critical role of EphA2 kinase-dependent activity promoting EWS angiogenesis.

CAV1 silencing in EWS cells results in loss of tumorigenicity, sensitization to chemotherapy and reduced migratory and invasive capabilities [42]. EphA2 appears to play a role in tumorigenesis in its non-phosphorylated state and possesses ligand-independent kinase activity *in vitro* [43], [44]. We did not observe significant changes in the malignant phenotype of cells transfected with the EphA2 dominant-negative construct (data not shown). However, we cannot discard the possibility that in EWS cells EphA2 might play a critical role in their tumorigenic phenotype independently of its kinase activity. Current studies in the laboratory are addressing this issue. Taken together, our results strongly suggest that the axis EphA2-CAV1 cause activation of AKT signaling to produce bFGF, promoting tumor induced endothelial cell migration and favoring EWS angiogenesis.

## Materials and Methods

### Ethics Statement

Animal care and procedures were followed according to the Institutional Guidelines for the Care and Use of Laboratory Animals. Ethics approval was provided by the locally appointed ethics committee from the Biomedical Research Institut (IDIBELL), Barcelona, Spain.

### Cell Culture, Stable Transfections and Stimulation Assays

A673, TC252 and STAET1 cell lines (gifts from Dr. Heinrik Kovar, Children's Cancer Research Institute, Kinderspitalgasse, Vienna, Austria [45]); RH1 cell line (gift from Dr. Peter Houghton, The Research Institute at Nationwide Children’s Hospital, Columbus, Ohio [46]); RDES, TC71 and SKES1 cell lines (bought from DSMZ, Leibniz Institute DSMZ-German Collection of Microorganisms and Cell Cultures, Braunschweig, Germany); and A4573 cell line (gift from Dr. Santiago Ramón y Cajal, Departament d’Anatomia Patològica, Hospital Universitari Vall d'Hebron-Universitat Autònoma de Barcelona, Barcelona, Spain [2]), all EWS cells, were cultured in RPMI 1640 (Invitrogen) supplemented with 10% heat-inactivated fetal bovine serum (Invitrogen). PAEC cells –Porcine Aortic Endothelial Cells– (gift from Dr. Cristina Costa, Institut d’Investigació Biomèdica de Bellvitge, l’Hospitalet de Llobregat, Barcelona, Spain [47]) were cultured in RPMI 1640 (Invitrogen) supplemented with 10% heat-inactivated bovine serum (Invitrogen) and endothelial mitogen (50 mg/l; Biomedical Technologies Inc.). All cell lines were incubated at 37°C in a humidified atmosphere of 5% CO_2_ in air. Exponentially growing cells within two sequential passages were used for all experiments. Cells were transfected using Fugene Transfection Reagent (Roche) or Lipofectamine 2000 (Invitrogen) following the protocols of the manufacturer. Transfected cells were selected with neomycin [0.4 mg/ml or 0.8 mg/ml, ShCAV1 [2], [16] and EphA2kd [41] (kindly gifted by Dr. Paola Chiarugi, University of Florence, Florence, Italy), respectively; Invitrogen] for 14 days, and antibiotic-resistant pools and individual colonies were isolated for further analysis and maintained in the presence of neomycin (0.4 mg/ml or 0.8 mg/ml). For studies using soluble ephrinA1, cells were stimulated with 5 µg/ml IgG/Fc or ephrinA1-Fc (R&D Systems) for 20 min. When needed AKT inhibitor IV (Merck Millipore) was used at a concentration of 5 µM.

### Immunoprecipitacion and Western Blot Analysis

EWS cells were lysed with radioimmunoprecipitation assay buffer (RIPA Buffer, Thermo Scientific) containing protease inhibitors (Complete, Mini; Protease Inhibitor Cocktail Tablets, Roche) and phosphatase inhibitors (PhosStop, Phosphatase Inhibitor Cocktail Tablets, Roche) and centrifuged at 13,000×*g*, at 4°C, for 30 minutes. The protein content of the supernatants was determined with the BCA assay system (Pierce). For immunoprecipitation, cells lysis was made using immunoprecipitation buffer with 50 mM Tris-HCl (pH 7.6), 150 mM NaCl, 1% NP-40 and containing protease and phosphatase inhibitors. Magnetic beads coated with Protein G (Millipore) were incubated following manufacturer’s protocol with previously incubated 500 µg protein samples with its respective antibody (CAV1 #610059 from BD Biosciences; EcK/EphA2 #05-480 and Phosphotyrosine #05-321 from Millipore, normal mouse IgG #sc-2025 and normal rabbit IgG #sc-2027 from Santa Cruz Biotechnology). After immunoprecipitation, beads were resuspended for denaturating elution in 30 µl of sample buffer. Lysate aliquots (50 µg) and immunoprecipitations were resolved by 8, 10 or 15% SDS-PAGE (depending on the size of the protein that was analyzed) and transferred onto nitrocellulose membranes (0.2 µm, Bio-Rad). After blocking with 5% skimmed milk in Dulbecco's PBS (DPBS) containing 0.1% Tween20 at room temperature for 1 hour, membranes were incubated overnight at 4°C with the appropriate primary antibody (CAV1; EcK/EphA2, FGF/basicFGF #05-118 and Phosphotyrosine from Millipore; FAK #3285, Akt #9272, Phospho-Akt Ser473 #4060 and Phospho-Akt-Thr308 #9275 from Cell Signaling Technology). Blots were then incubated at room temperature for 1 hour with a horseradish peroxidase-conjugated secondary antibody (goat anti-rabbit and goat anti-mouse) and the peroxidase activity was detected by enhanced chemiluminescence (Pierce) following the instructions of the manufacturer. Immunodetection of β-actin (#ab49900 from Abcam) was used as a loading reference.

### Proliferation Assay

A density of 5×10^4^ cells per well was seeded on 12-well-plate overnight in complete culture medium and then treated with RPMI alone (Negative control) or CM from TC71 and RDES models for a 3-day incubation. The cell numbers were measured every 24 hours by the trypan blue exclusion assay.

### Transwell Migration Assay

PAEC cells (2.0×10^4^) in 100 µL of serum-free medium were added to the top chamber of 8 µm Costar polycarbonate membrane transwell (Transwell Permeable Supports, Corning), while 500 µL of CM from EWS cell models was added to the bottom chamber. CM was obtained by culturing cells for 48 hours in fresh RPMI media without serum. After this time media was centrifuged and filtered to avoid any suspended cells. After allowing migration for various times (37°C, 100% humidity, 5% CO_2_ in air), cells on the upper membrane surface were removed and migrant cells on the membrane underside were fixed using 70% ethanol, stained using 0.1% crystal violet solution (Sigma-Aldrich), and visualized and counted under the microscope. Neutralizing antibody against bFGF (Anti-FGF2/basic FGF #05-117) and human recombinant FGF-2/basic (FGF2 #01-106) were from Millipore and were used at mentioned concentrations. Data were presented as the average number of migrating cells in 5 high-power fields (x200). Each experiment was done in triplicate, and then the data were averaged for statistical analysis.

### *In vivo* Studies

Experiments to evaluate the tumor development of CAV1 knocked down models were carried out using immunodeficient female (5–6 weeks old) athymic nude mice purchased from Harlan Laboratories. Mice were injected subcutaneously (s.c) into the right posterior flank with 3×10^6^ RDES (n = 20), TC71 (n = 20) and SKES1 (n = 20) low CAV1 models and RDES (n = 10) and TC71 (n = 10) EphA2-kd models in 100 µl of RPMI (Invitrogen). Once tumors reached a mean volume of about 1 cm^3^ (21 days), mice were euthanized. Tumors were fixed in formaldehyde at 4% and embedded in paraffin. Tumor volumes were calculated by the formula *V* = (1/2) *a*×*b* ^2^, where *a* is the longest tumor axis, and *b* is the shortest tumor axis. Data are given as mean ± SD. Statistical analysis was done by an unpaired Student's *t* test.

### Immunohistochemistry and Immunofluorescence

Immunohistochemical techniques were done essentially as previously described [2]. Expression of CAV1 in human EWS and xenografts was analyzed using a rabbit polyclonal antibody (CAV1 #610059). Microvascular density was analyzed using a polyclonal antibody for CD31 (Platelet Endothelial Cell Adhesion Molecule-1: PECAM-1) from Abcam (#ab28364). Data were presented as the average number of positive vessels in the whole section of the tumor from all samples comparing the model. EphA2 expression was analyzed using a polyclonal antibody for EphA2 (EphA2 IHC Antibody from Bethyl Laboratories #IHC-00427). For immunofluorescence, EWS cells were cultured in sterile slides (Millicell EZ slide from Millipore) for 24 hours, fixed with 4% formaldehyde for 30 minutes, washed thrice in Dulbecco's PBS (DPBS), permeabilized in 0.1% Triton X-100 (Sigma) for 2 minutes, blocked for 1 hour in blocking buffer (10% goat serum in DPBS) and incubated with primary antibodies overnight. Cells were then washed thrice in DPBS for 5 minutes each followed by a 1 hour incubation with secondary antibodies (Alexa Fluor 488 goat anti-mouse and Alexa Fluor 594 goat anti-rabbit; Invitrogen). Then, cells were washed twice in DPBS for 10 minutes and twice in distilled water for 10 minutes, and mounted in ProLong Gold antifade reagent with 4′,6′-diamidino-2-phenylindole (Invitrogen). Photographs were taken with a Leica TCS SP5 spectral confocal microscope (argon, 405 diode and DPSS561 lasers) using a lambda blue 63×1.35 numerical aperture oil objective. Images were analyzed with the MacBiophotonics Program. CAV1 and EphA2 expression in EWS cells was analyzed by using the same specific antibodies. Expression of Phospho-FAK was detected in PAEC cells using a polyclonal antibody for Phospho-FAK –Tyr397– #3283 from Cell Signalling. PAEC cells, serum depleted for other 16 hours were cultured in conditioned media from the TC71/ShCAV1 model for 48 hours.

### Reverse Transcription-PCR (RT-PCR)

Total RNA (2 µg), extracted using the Total RNA Isolation Kit (NucleoSpin RNA II, Macherey-Nagel), was used for cDNA synthesis with SuperScript II Reverse Transcriptase (Invitrogen). Primers 5′-CGGAATGAGGACTACACCATACATGTGCAGC-3′ (forward) and 5′-AAGCAGCGGTCTTCATGCTGGTGGATGGGTT-3′ (reverse) were used for amplification of *Ephrin-A1* (326 bp); and for *β-Actin* (432 bp), 5′-CGGGACCTGACTGACTACCTC-3′ (forward) and CTTCATTGTGCTGGGTGC (reverse). Amplification of *Ephrin-A1* was adjusted at an annealing temperature of 58°C and 59.5°C for *β-Actin*. Amplifications of *bFGF*, *PDGF-A*, *PDGF-B*, *MK*, *TGFB* and *VEGF* were carried out using specific primers (Table S1). For each set of primers, the number of cycles was adjusted so that the reaction end points fell within the exponential phase of product amplification, thus providing a semi-quantitative estimate of relative mRNA abundance. RT-PCR determinations were carried out thrice for each relevant transcript. Primers were from Invitrogen.

### Tissue Samples

28 tumor samples were procured from the archives of the Tumor banks of St. Joan de Deu Hospital (13 patients) and Centro de Investigación del Cancer-IBMCC (15 patients) Pathology Departments and referring institutions. All tumor samples were fixed in formalin, embedded in paraffin, and had not undergone decalcification. Associated clinical data were available only for the St. Joan de Deu Hospital. Because all patients showed EphA2 expression no clinical correlations could be made.

### Statistical Analysis

Data were analyzed for statistical significance using Student's *t* test. In the case of confocal images pair wise correlations were evaluated by the two-tailed Pearson-test, measuring the strength of the association between two variables. For immunoblot and RT-PCR experiments relative expression of each gene to the reference gene (Actin) was calculated measuring each band area after subtracting background using Image J software (IJ 1.46r, NIH, USA). Unless otherwise stated, experiments were performed thrice and *P*≤0.05 was regarded as significant.

## Supporting Information

Figure S1 ***bFGF*** **expression in EWS cell lines.** Several growth factors (*PDGF-A*, *PDGF-B*, *MK*, *TGFB1*, *VEGF* and *bFGF*) were analyzed by RT-PCR.
(TIF)

Figure S2 **CAV1 silencing reduces** ***bFGF*** **expression.** Several growth factors (*PDGF-A*, *PDGF-B*, *MK*, *TGFB1*, *VEGF* and *bFGF*) were analyzed by RT-PCR in CAV1 knocked down models. Results showed that *bFGF* was the only one reduced constantly in all models.
(TIF)

Figure S3 **EphA2 protein expression in human tumor samples.** Immunohistochemical analysis of positive EWS samples demonstrating the presence of EphA2 (left panel) and the absence of other members of the family (EphB3 and EphB4). EphA2 expression was positive in all tumors and the pattern of expression was observed in both membrane and cytoplasm. Scale bar, 50 µm.
(TIF)

Figure S4 **RT-PCR for** ***ephrin-A1*** **.** EphA2 most common ligand, *ephrin-A1,* is expressed in all EWS cells tested.
(TIF)

Figure S5 **Co-expression of CAV1 and EphA2 proteins in mouse xenografts samples.** Co-Immunofluorescence of CAV1 and EphA2 showing co-localization in cell membrane in paraffin embedded tissue from mouse xenograft. Scale bar, 20 µm.
(TIF)

Figure S6 Hematoxylin and Eosin (H&E) staining in EphA2-Kd paraffin-embedded xenografts from RDES and TC71 models showing a decrease in tumor volume and an increase of necrosis (*) in EphA2-Kd tumors. Scale bar, 50 µm.
(TIF)

Table S1 **Table of primers used in amplification of pro-angiogenic growth factors.**
(TIF)
