# Supplementary figures and images for: EphA2-Induced Angiogenesis in Ewing Sarcoma Cells Works through bFGF Production and Is Dependent on Caveolin-1
Source: PLoS One. 2013 Aug 12;8(8):e71449. doi: 10.1371/journal.pone.0071449 (PMC3741133; doi:10.1371/journal.pone.0071449)

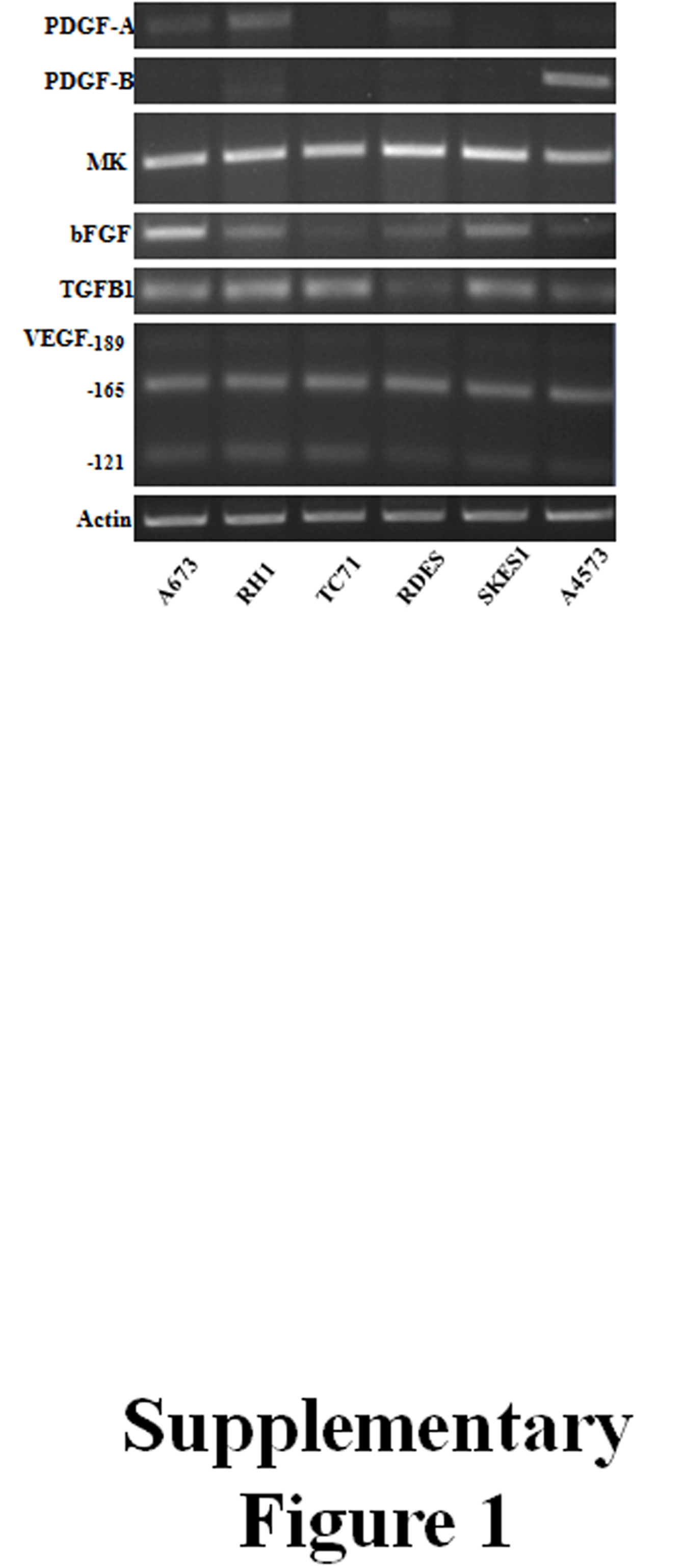

Supplement: Figure S1 — bFGF expression in EWS cell lines. Several growth factors (PDGF-A, PDGF-B, MK, TGFB1, VEGF and bFGF) were analyzed by RT-PCR. (TIF) [file pone.0071449.s001.tif]

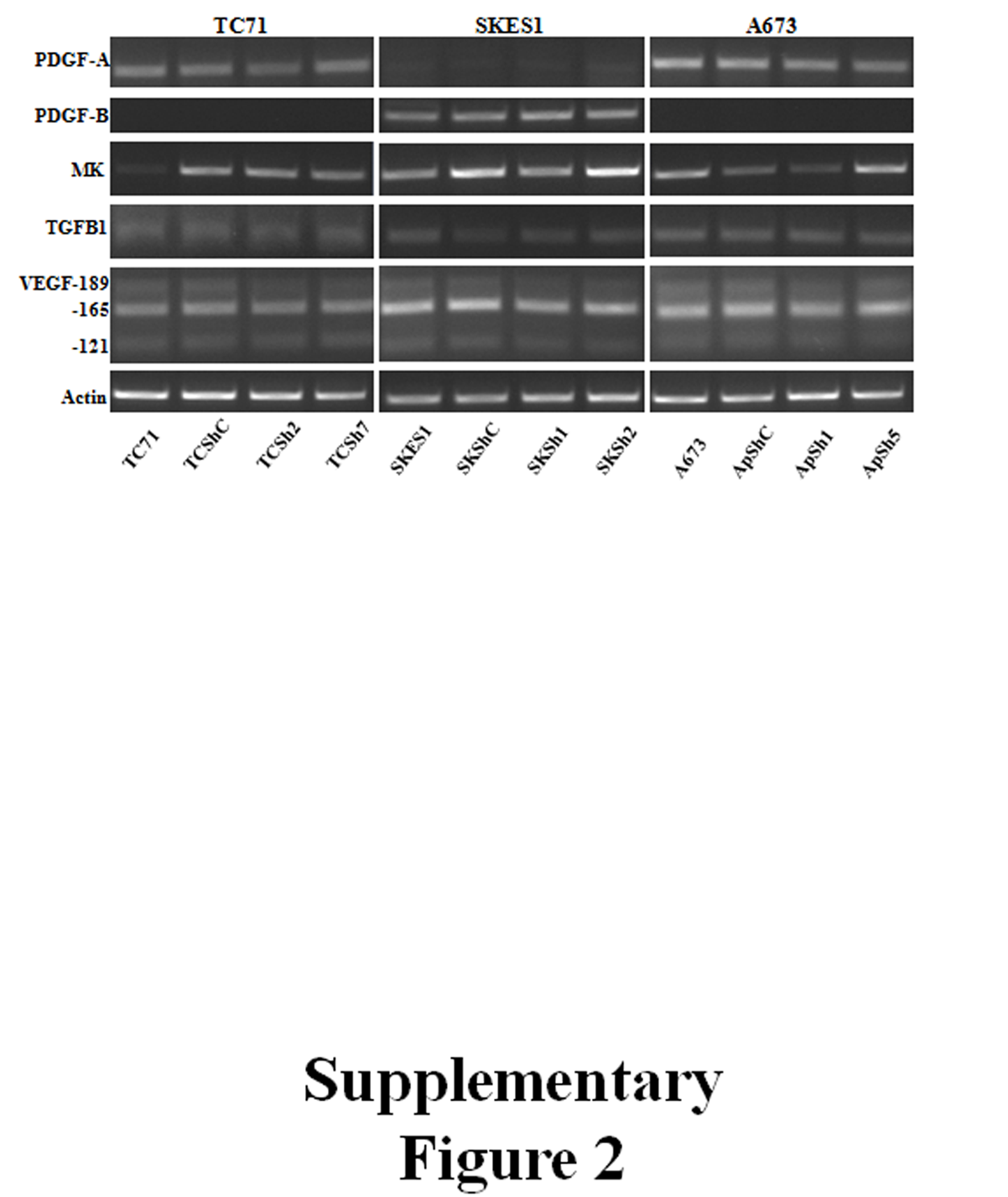

Supplement: Figure S2 — CAV1 silencing reduces bFGF expression. Several growth factors (PDGF-A, PDGF-B, MK, TGFB1, VEGF and bFGF) were analyzed by RT-PCR in CAV1 knocked down models. Results showed that bFGF was the only one reduced constantly in all models. (TIF) [file pone.0071449.s002.tif]

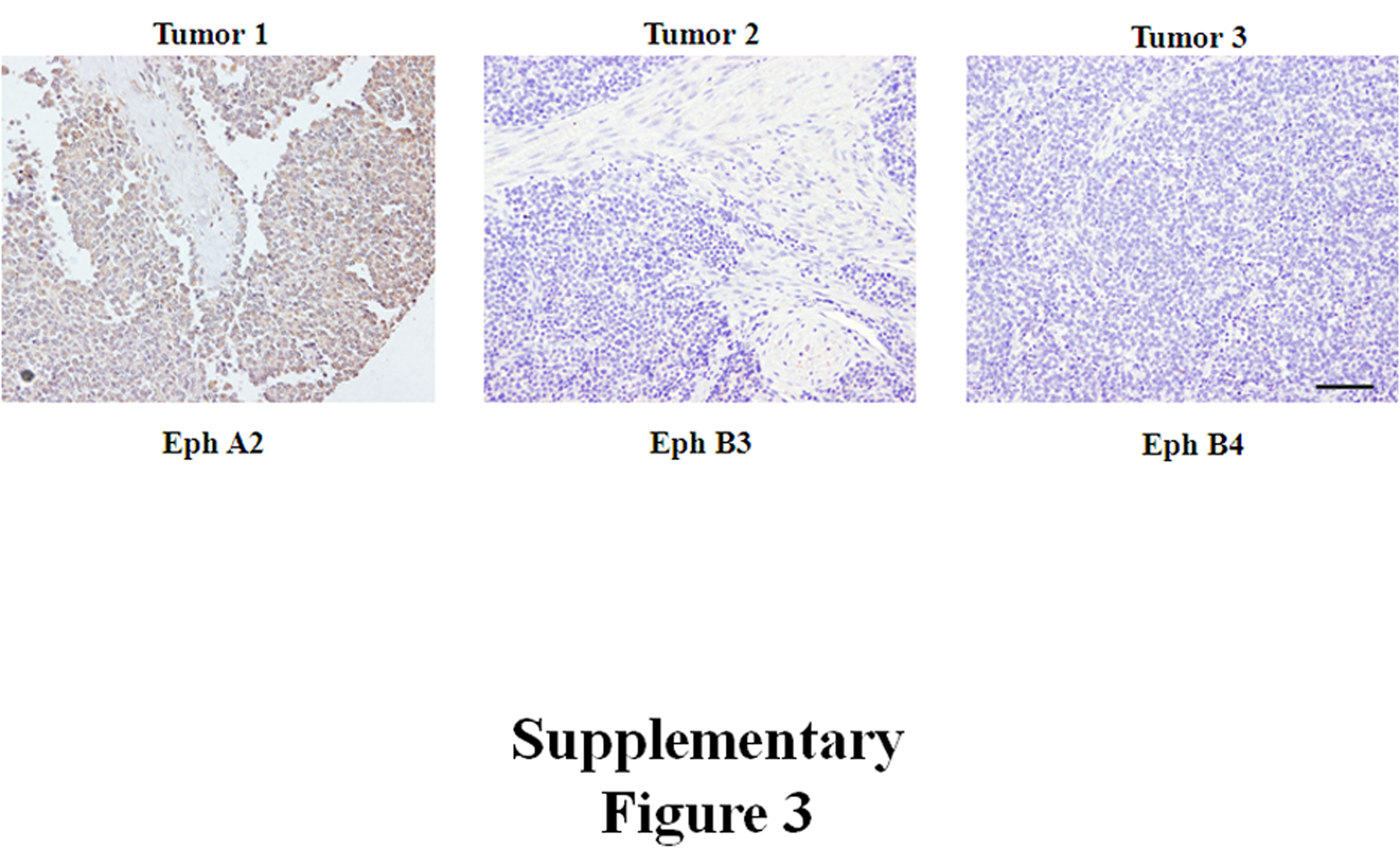

Supplement: Figure S3 — EphA2 protein expression in human tumor samples. Immunohistochemical analysis of positive EWS samples demonstrating the presence of EphA2 (left panel) and the absence of other members of the family (EphB3 and EphB4). EphA2 expression was positive in all tumors and the pattern of expression was observed in both membrane and cytoplasm. Scale bar, 50 µm. (TIF) [file pone.0071449.s003.tif]

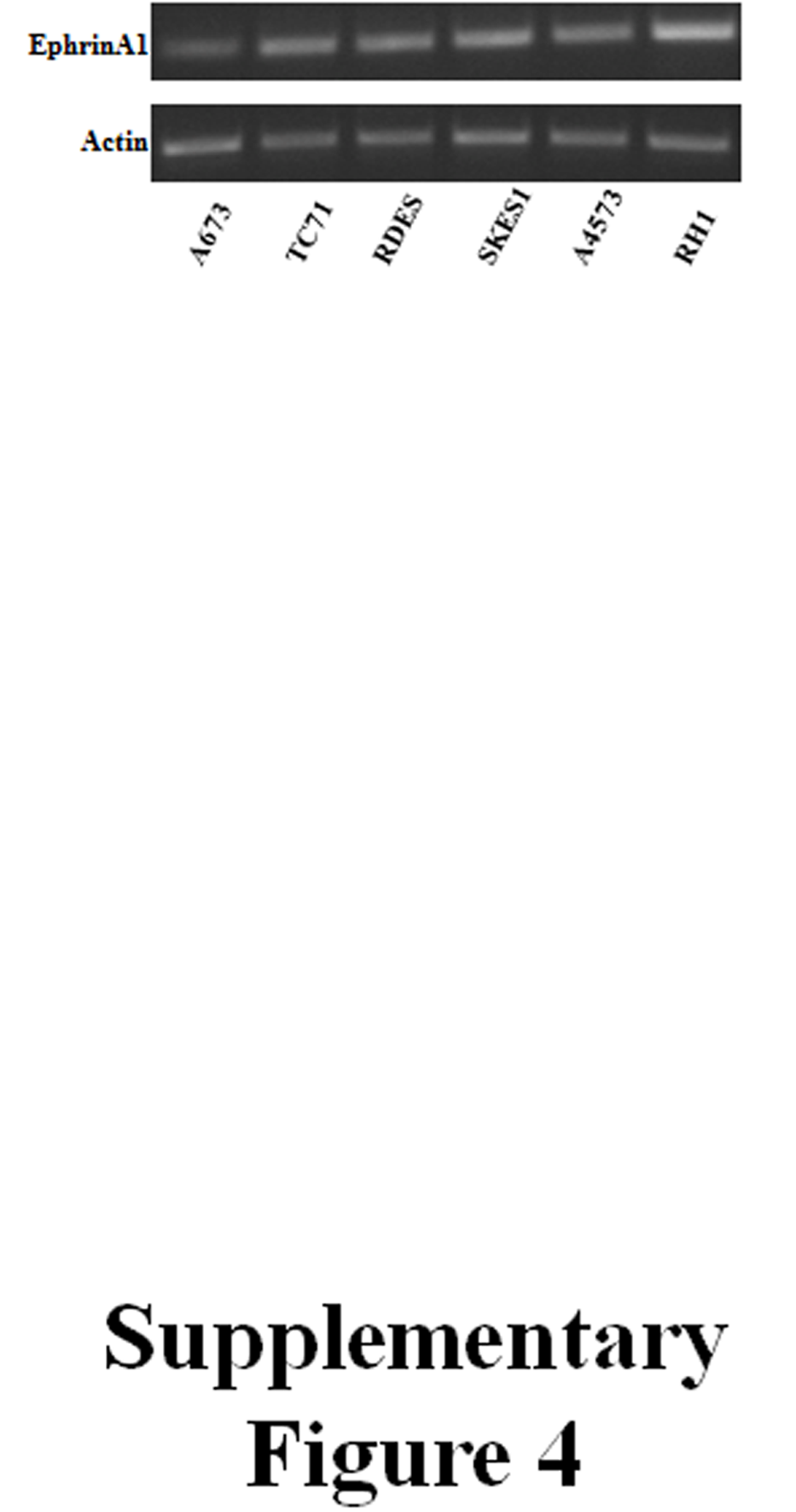

Supplement: Figure S4 — RT-PCR for ephrin-A1 . EphA2 most common ligand, ephrin-A1, is expressed in all EWS cells tested. (TIF) [file pone.0071449.s004.tif]

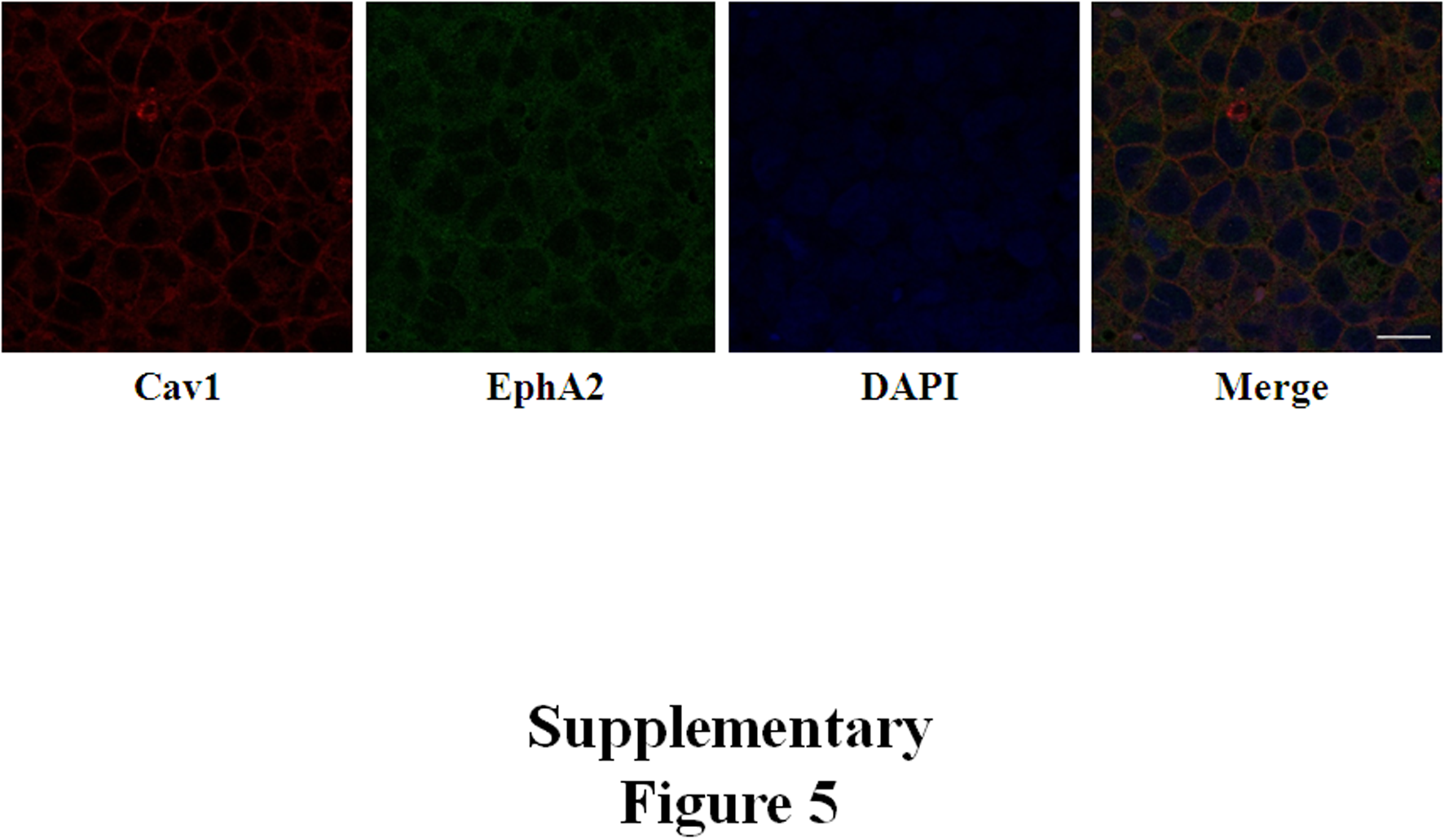

Supplement: Figure S5 — Co-expression of CAV1 and EphA2 proteins in mouse xenografts samples. Co-Immunofluorescence of CAV1 and EphA2 showing co-localization in cell membrane in paraffin embedded tissue from mouse xenograft. Scale bar, 20 µm. (TIF) [file pone.0071449.s005.tif]

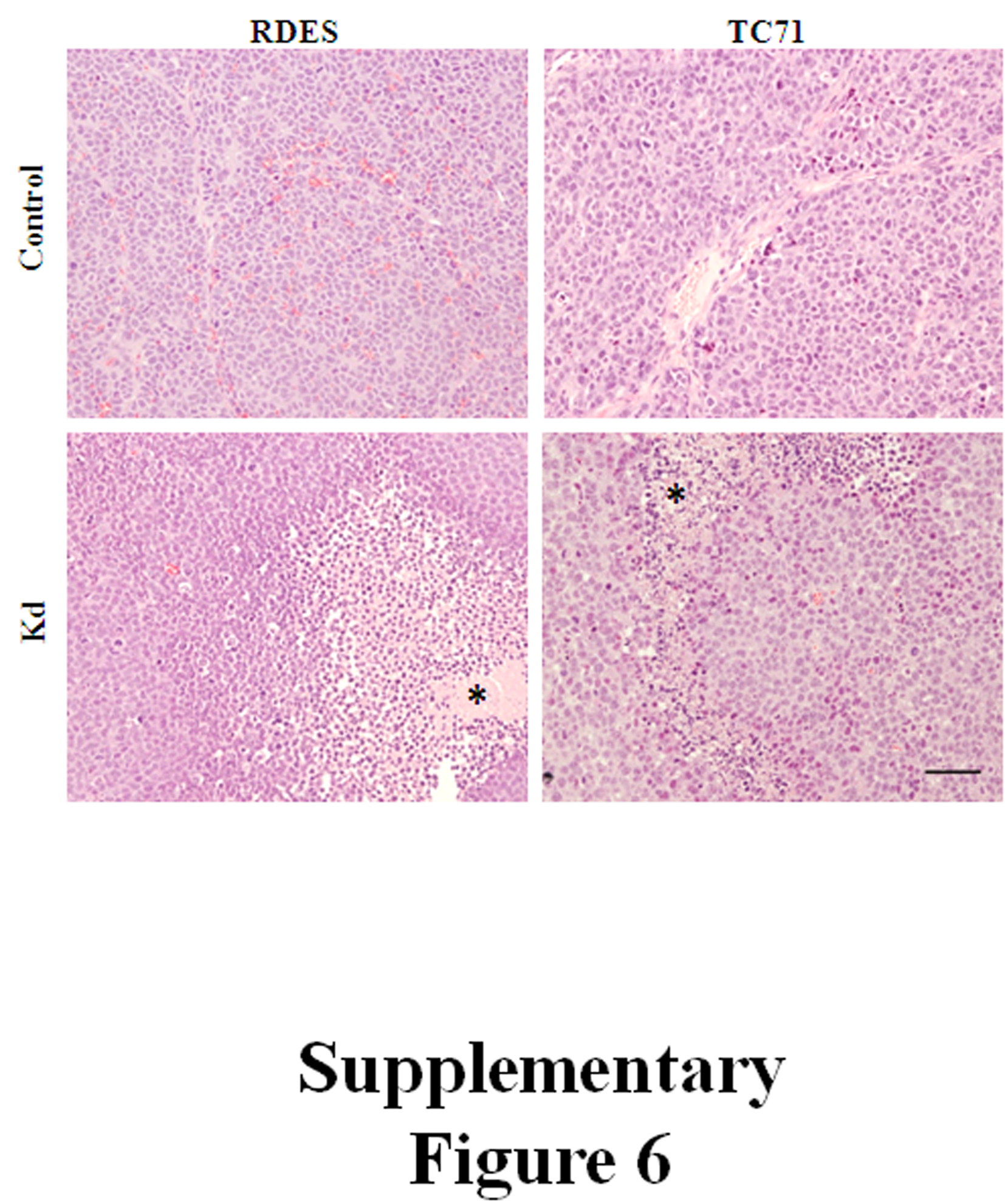

Supplement: Figure S6 — Hematoxylin and Eosin (H&E) staining in EphA2-Kd paraffin-embedded xenografts from RDES and TC71 models showing a decrease in tumor volume and an increase of necrosis (*) in EphA2-Kd tumors. Scale bar, 50 µm. (TIF) [file pone.0071449.s006.tif]

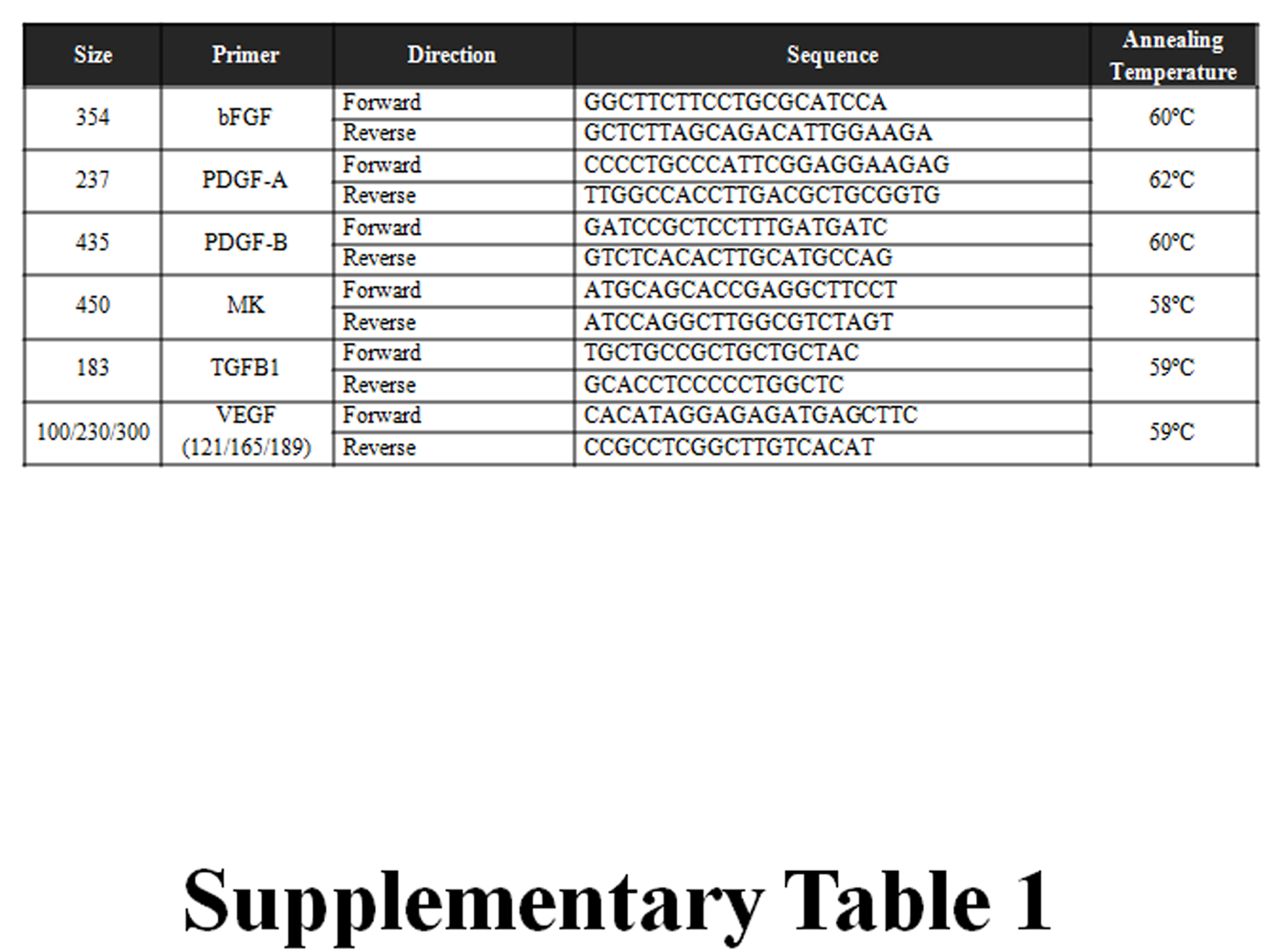

Supplement: Table S1 — Table of primers used in amplification of pro-angiogenic growth factors. (TIF) [file pone.0071449.s007.tif]
